# Effect of bednets and indoor residual spraying on spatio-temporal clustering of malaria in a village in South Ethiopia: a longitudinal study

**DOI:** 10.1186/1475-2875-11-S1-P66

**Published:** 2012-10-15

**Authors:** Eskindir Loha, Bernt Lindtjørn

**Affiliations:** 1 School of Public and Environmental Health, Hawassa University, Hawassa, Ethiopia; 2 Centre for International Health, University of Bergen, Bergen, Norway

## Background

Understanding the spatio-temporal pattern of malaria transmission where prevention and control measures are in place would help fine-tuning of strategies. The objective of this study was to assess the effect of mass bednets distribution and indoor residual spraying with insecticides on spatio-temporal clustering of malaria in one malaria endemic village in south Ethiopia.

## Methods

A longitudinal study was conducted from April 2009 to April 2011. The study population was 6631 in 1388 households. We used active and passive searches for malaria cases for 101 weeks. A software for spatial and space-time statistics, SatScan v9.1.1 was used to identify statistically significant retrospective space-time clusters. A discrete Poisson based model was applied with an aim to get areas with high rates. PASW Statistics 18 was used to analyze individual level factors using generalized Poisson loglinear model.

## Results

The total number of all types of malaria episodes was 622 resulting 45.1 episodes per 1000 per year; of which, episodes of *Plasmodium Falciparum* and *Vivax* was 316 (22.9 per 1000 per year) and 306 (22.2 per 1000 per year), respectively. Indoor residual spraying with DDT (and later with Deltamethrin) and free mass insecticide treated nets distribution were carried out during the study period. There was space-time clustering of malaria episodes at a household level. The spatio-temporal clustering of malaria was not influenced by free mass insecticide treated nets distribution, however, the time-span of the spatio-temporal clustering of malaria ended after indoor residual spraying with Deltamethrin. The reason for having the clusters on the east-south edge of the village was consistent with the finding showing increased risk of acquiring malaria infection for individuals living nearer to the identified vector breeding place.

## Conclusion

The risk of getting malaria infection varied significantly within one village. Free mass insecticide treated nets distribution did not reduce spatio-temporal clustering of malaria, but indoor residual spaying affected malaria clustering in the village.

